# Deposition of Sm-Co Coatings by Chronoamperometric Method

**DOI:** 10.3390/ma19112318

**Published:** 2026-05-31

**Authors:** Hubert Kamiński, Katarzyna Skibińska, Dawid Kutyła, Mateusz Marzec, Aun Nawaz Khan, Piotr Żabiński

**Affiliations:** 1 Department of Physical Chemistry and Metallurgy of Non-Ferrous Metals, Faculty of Non-Ferrous Metals, AGH University of Krakow, Al. Adama Mickiewicza 30, 30-059 Krakow, Poland; kskib@agh.edu.pl (K.S.); kutyla@agh.edu.pl (D.K.); khanaunnawaz@gmail.com (A.N.K.); zabinski@agh.edu.pl (P.Ż.); 2 Academic Centre for Materials and Nanotechnology, AGH University of Krakow, Al. A. Mickiewicza 30, 30-059 Krakow, Poland

**Keywords:** electrodeposition, samarium-cobalt alloys, rare-earth metals, chronoamperometry, complexing agent, L-arginine, glycine

## Abstract

The subject of this study is the electrochemical synthesis of samarium–cobalt (Sm-Co) alloy coatings on a copper substrate from aqueous solutions using chronoamperometric methods. The study focused on assessing the effect of ecological complexing agents—L-arginine and glycine—on the deposition kinetics and quality of the deposits obtained within a potential range of −1.1 V to −1.8 V vs. Ag/AgCl. Morphological analyses indicated that the type of amino acid used determines the layer growth mechanism. It was found that exceeding the potential of −1.4 V results in a rapid increase in samarium content in the alloy, reaching maximum values of 29 at.% for the system with L-arginine and 35 at.% for the system with glycine at a potential of −1.8 V. X-ray Diffraction (XRD) structural studies confirmed the successful synthesis of the Co_8.5_Sm intermetallic phase directly by electrodeposition, while X-ray Photoelectron Spectroscopy (XPS) analyses indicated the presence of oxides and hydroxides on the deposit surface. Despite obtaining a high samarium content, it was observed that intense hydrogen co-evolution at low potential leads to a decrease in current efficiency and the formation of internal stresses and cracks in the structure of the coatings.

## 1. Introduction

Electrodeposition, also known as electroplating, is a method of depositing a metal or an alloy on the surface of a substrate using electrochemical means [1,2]. It is a process of film growth occurring through the electrochemical reduction of metal ions from an electrolyte [3]. The electrodeposited material possesses a wide range of properties as compared to the base material. Depending on the application, these properties can vary and can influence aesthetics, wear and corrosion resistance, adhesion, and anti-friction properties, etc. [4,5]. A classic example of electrodeposition is the Zn-Cu system [6,7]. Standard cathodic mechanisms [8] and anode configurations [9,10], including copper anodes [11] and various alloy systems [12,13,14,15,16], are commonly utilized. However, in comparison to metal and alloy deposition, rather limited research is conducted on the electrodeposition of rare-earth elements, particularly using aqueous electrolytes [17]. According to Arrachart et al., the reduction potential of rare-earth elements (REEs) is negative to such an extent that hydrogen evolution becomes inevitable [18].

The rare-earth elements (REEs) [19,20] are strategically important across high-technology sectors due to their unique chemical and magnetic properties [21]. Therefore, it is desirable to develop cost-effective and environmentally friendly electrochemical techniques for depositing rare-earth elements on commonly available substrates, e.g., copper. While traditional methods of obtaining Sm-Co films include vacuum-based techniques like sputtering or evaporation [22,23], electrodeposition is gaining attention as a more cost-effective and versatile alternative, allowing for the fabrication of complex micro-structures [24]. Specifically, chronoamperometric deposition offers superior control over film growth and morphology, resulting in higher-quality layers than traditional techniques [25,26].

As established before, the use of aqueous solutions for the electrodeposition of rare-earth elements on metallic substrates is challenging due to their highly negative reduction potentials [27,28]. For instance, the reduction potential of samarium exceeds −2.3 V [29], a level at which strong hydrogen evolution significantly increases the local pH at the cathode [30]. This results in the formation of hydroxide on the metal–coating interface, hampering further electrodeposition of Sm [31,32,33]. Hydrogen evolution during metal or alloy deposition may lead to a gradual loss in the mechanical properties of the deposit [34]. To mitigate such degradation, carbon can be incorporated into the alloy, as it effectively reduces corrosion and improves the chemical stability of the layers [35]. However, it is still possible to deposit rare-earth elements from an aqueous electrolyte by selecting appropriate complexing agents. Jundhale et al. electrodeposited samarium using a tartarate bath [36]. The process involved adding 50 mM Sm_2_O_3_ to water after adding 0.1–1 M tartaric acid as a complexing agent. The authors electrodeposited samarium films at varying potentials ranging from approximately −0.455 V to −1.19 V versus SCE, depending on the substrate. Wei et al. electrodeposited an alloy with a 0.15 M concentration of glycine as a complexing agent [17]. Researchers experimented with a different potential range from −0.6 V to −3.4 V vs. SCE.

Another amino acid that is used as a complexing agent is cysteine. It consists of a carboxylic group and a thio-group, which, apparently, are responsible for the loss of properties when used as a complexing agent [37,38,39,40]. Glycine consists of a single carboxylic group. Research work indicates that it has been widely used as a complexing agent. Specifically, glycine has been utilized for the successful electrodeposition of iron-group rare-earth alloys (such as Fe-RE) from aqueous chloride or sulfamate-based solutions. On the other hand, L-arginine represents an interesting, easily available, and eco-friendly alternative in the amino acid domain [41,42]. Additionally, arginine (C_6_H_14_N_4_O_2_) can be added to electrolytes as both a complexing agent and a carbon precursor for the synthesis of alloys. L-arginine addition (0.1 M) to the electrolyte for Co-Mo deposition in the magnetic field smoothed the alloy’s surface, probably by lowering the amount of co-evolved hydrogen [43]. Co-W-C alloys electrodeposited from the bath containing the addition of arginine show improved catalytic properties in the hydrogen evolution process [44]. In the case of Ni-C alloys, with the increase in arginine concentrations, the amorphization of the nickel crystalline structure progressed, and the reduction in average crystallite size was observed [45]. However, its application as a stabilizing and complexing agent specifically for rare-earth-containing systems-such as the co-deposition of Sm-Co alloys from aqueous baths-remains unexplored [46,47,48,49]. An additional aspect of the research is the assessment of the effect of carbon, which improves, among other things, corrosion resistance on the morphology of the obtained layer and its durability [35,50,51,52,53,54].

Despite the growing demand for Sm-Co thin films, no studies have been published on the use of L-arginine to stabilize aqueous baths specifically for the co-deposition of rare-earth transition-metal alloys, the properties of the resulting layer, or the effect of carbon addition on its properties. The innovativeness of this research is centered on the pioneering use of L-arginine as an ecological complexing agent and a carbon precursor specifically for the aqueous electrodeposition of rare-earth transition-metal alloys, enabling the direct synthesis of the Co_8.5_Sm intermetallic phase from an aqueous solution. While glycine has been previously employed for Sm-Co co-deposition by Wei et al. [17], a systematic side-by-side comparison between these two amino acids under identical experimental conditions has not been conducted. This work addresses this gap by presenting a rigorous comparative study between the newly proposed L-arginine bath and the established glycine bath, providing new insights into their respective effects on electrolyte stability, deposition kinetics, and film morphology.

The primary goal of this investigation is to systematically evaluate the effectiveness of L-arginine and glycine in bath stabilization and determine their precise influence on the structural and compositional properties of the deposited Sm-Co layers. Specifically, the study aims to analyze the electrochemical kinetics of the co-deposition process, determine the current efficiency and elemental composition via X-ray Fluorescence (XRF) and Energy-Dispersive X-ray Spectroscopy (EDS), characterize the surface morphology and phase development using Scanning Electron Microscopy (SEM) and X-ray Diffraction, and identify chemical states on the surface using X-ray Photoelectron Spectroscopy. Although Sm-Co thin films are highly sought after for magnetic microdevices and navigating system applications, the scope of this study is strictly limited to the fundamental physical chemistry, electrochemistry, and materials characterization of the deposition process, leaving magnetic performance optimization for future investigations. This sequence of goals directly translates into the experimental results presented in the subsequent sections of this manuscript.

## 2. Research Methodology

The components of the electrolyte for the deposition of Sm-Co alloys were Sm_2_O_3_ (Sigma-Aldrich, St. Louis, MO, USA), CoCl_2_ ·6H_2_O (Alfa Aesar, Haverhill, MA, USA), and a complexing agent in the form of L-arginine (Thermo Fisher Scientific, Waltham, MA, USA) or glycine (Thermo Fisher Scientific, Waltham, MA, USA). Samarium oxide does not dissolve in distilled water at room temperature. Increased solubility of the Sm_2_O_3_ compound in distilled water was achieved by lowering the pH of the solution to 1 using hydrochloric acid (HCl) (Chempur, Piekary Śląskie, Poland). In the next step, 0.1 M CoCl_2_ was introduced into the solution, followed by the addition of 0.2 M L-arginine/glycine, acting as the complexing agent. The addition of the complexing agent caused the pH of the solution to increase to 3.2. It was observed that leaving the solution without stirring for 24 h leads to the reprecipitation of Sm_2_O_3_. To maintain the stability of the solution, a few drops of HCl were added again.

**Figure 1:**
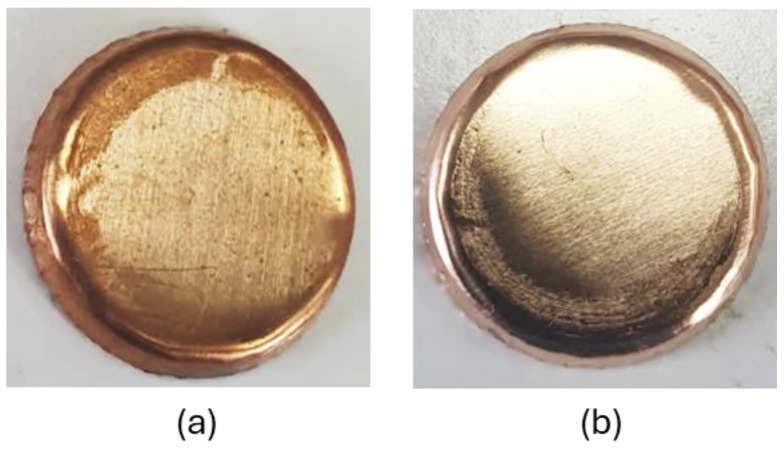
Copper substrate surface (**a**) before etching and (**b**) after etching.

The coatings were deposited using chronoamperometry in a three-electrode system, with a copper substrate as a working electrode, platinum mesh as a counter, and a silver chloride Ag/AgCl reference electrode. Copper surface preparation involved 10 s etching in a mixed solution of concentrated acetic (95,9%), phosphoric (36%), and nitric acids (65%) (mixed in a controlled 1:1:1 ratio) at 95 °C (Figure 1).

The process was completed by rinsing in distilled water and drying with hot air. The samples were weighed before and after the electrodeposition process, and the obtained mass difference was compared with values associated with the function of potential or solution aging time. The deposited surface was 1 cm in diameter. Surface morphology was determined using scanning electron microscopy using a JCM-6000 Plus instrument (JEOL, Tokyo, Japan), operating at 750× magnification and a 10 kV electron beam spread, which allowed for layer control and crack initiation by user-induced stress extensions. The chemical composition of the Sm-Co alloy and the content of individual elements as a function of the applied application or its removal time were analyzed by XRF using a Rigaku Primini spectrofluorometer (Rigaku, Tokyo, Japan). Crystal structure analysis, along with analytical phase identification, was performed by XRD using a Rigaku MiniFlex II diffractometer (Rigaku Corporation, Tokyo, Japan) equipped with a Cu Kα (λ = 1.54059 Å) radiation source. XPS analysis was performed on 0.2 M L-arginine and glycine samples to characterize their surface composition. The XPS analyses were carried out in a PHI VersaProbeII Scanning XPS system (PHI, Chigasaki, Japan) using monochromatic Al Kα (1486.6 eV) X-rays focused to a 100 μm spot and scanned over the area of 400 μm × 400 μm. The photoelectron take-off angle was 45°, and the pass energy in the analyzer was set to 46.95 eV (0.1 eV step) to obtain high-energy-resolution spectra for the C 1s, O 1s, Co 2p, Sm 3d, N 1s, and Cl 2p regions. A dual beam charge compensation with 7 eV Ar^+^ ions and 1 eV electrons was used to maintain a constant sample surface potential regardless of the sample conductivity. All XPS spectra were charge referenced to the unfunctionalized, saturated carbon (C-C) C 1s peak at 284.8 eV. The operating pressure in the analytical chamber was less than 4 × 10^−9^ mbar. Deconvolution of spectra was carried out using PHI MultiPak software (v.9.9.3). The spectrum background was subtracted using the Shirley method.

Within the experiment geometry, the information depth of analysis was about 5 nm. The Co 2p3/2 spectra were fitted with five lines, with the first line centered at 780.8 eV which may indicate the presence of Co^2+^ type compounds like CoO, CoCl_2_ or Co(OH)_2_ all lying at the same binding energy range [55,56,57,58]. The lines within the energy range of 782–790 eV are due to the multiplet splitting phenomena and additionally prove the Co^2+^ oxidation state presence. The Sm 3d5/2 spectra were fitted with two lines: the first minor found at 1080.7 eV and the second major line centered at 1083.5 eV both originating from the presence of Sm^3+^ oxidation state. Two lines for one chemical state come from multiplet splitting phenomena for Sm^3+^ compounds [59,60,61]. The O 1s spectra were fitted with three components: first line centered at 529.4 eV, which indicates mainly lattice oxygen in oxides (O-Sm, O-Co), second line at 531.3 eV indicating defective oxygen in metal oxides and/or organic species containing O=C type bonds, and third line at 533.0 eV from either metal hydroxides and/or some part of O-C organic bonds [6,7,8,60,61,62]. The Cl 2p spectra were fitted with a doublet structure (doublet separation p3/2–p1/2 equals 1.6 eV) with the main 2p3/2 line centered at 198.4 eV, which indicates the presence of Cl^−^ ions in chlorides [62,63]. The C 1s spectra were fitted with five components. First line found at 284.8 eV indicates aliphatic carbon, second line at 286.0 eV points out the existence of C-O and/or C-N groups, third line at 287.2 eV indicates C=O and/or O-C-O and/or C=N groups, fourth line found at 288.6 points to O-C=O type bonds, and last line centered at 289.9 eV indicates O-(C=O)-O type bonds [63]. The N 1s spectra were fitted with three lines: the first line positioned at 398.2 eV indicating organic type nitrogen, e.g., amines, the second line found at 399.6 eV comes from C=N bonds, and the third line centered at 401.2 eV which shows NH^4+^ ions presence or NO_2_^−^ groups [62,63].

The influence of various factors on the electrodeposition process was investigated, including complexing agent (electrolyte I–II) and aging times of 1, 2, 3, 4, and 7 days for both solutions. The electrodeposition of the aged solutions was performed at a potential of −1.5 V vs. Ag/AgCl.

The experimental parameters are summarized in Table 1:

Electrochemical measurements were performed using a BioLogic SP-300 potentiostat (BioLogic Sciences Instruments, Seyssinet-Parise, France), controlled by dedicated EC-Lab software (version 11.50). The experiments were conducted in a dedicated electrochemical cell made of polytetrafluoroethylene. The system potential was controlled relative to a silver chloride Ag/AgCl reference electrode. For each experiment, the deposition time was constant—30 min.

The current efficiency of the process was determined based on the ratio of the theoretical charge, calculated using Faraday’s law, to the actual charge recorded during chronoamperometric tests:

(1) $$\%CE=\frac{Q\;theoretical}{Q\;experimental}\times 100\%$$

where the theoretical charge Q was defined as:

(2) $$Q=\frac{m\times n\times F}{M}$$

In the above equations, m (g) denotes the mass of the deposited coating, n is the number of electrons participating in the reaction, F is the Faraday constant (96.485 C/mol), and M (g/mol) is the molar mass of the deposited material.

## 3. Results

**Figure 2:**
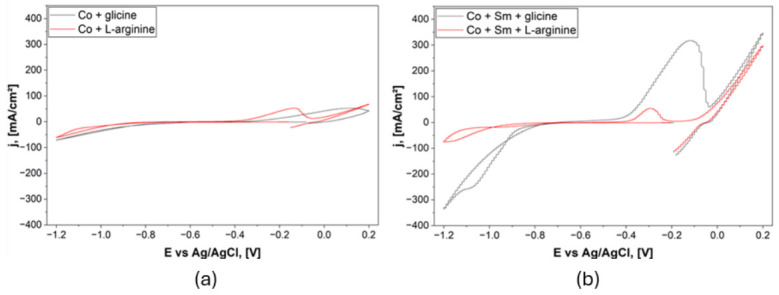
Cyclic voltammetry curves for solutions containing: (**a**) cobalt and a complexing agent, (**b**) cobalt, samarium, and a complexing agent.

The use of various complexing agents affects the stability of the solution and, consequently, the quality of the deposited coatings. This work investigated the effect of L-arginine and glycine on alloy deposition. In the next part of this work, the influence of solutions’ aging on the deposited layer was examined. Before starting the electrodeposition process, cyclic voltammetry curves were measured (Figure 2).

Comparative analysis of voltametric curves recorded in two- and three-component systems indicates the role of interactions between metal ions and complexing agents in the electrodeposition process. In the system containing only cobalt(II) ions in the presence of glycine and L-arginine, the CV curves are characterized by low current densities not exceeding 100 mA/cm^2^. In the cathodic region, at potentials more negative than −0.8 V, the onset of Co^2+^ ion reduction is observed, while in the return cycle, anodic peaks ranging from −0.3 V to 0.1 V are recorded, indicating limited efficiency of pure metal deposition under the given electrolysis conditions. The situation changes after the introduction of samarium(III) ions to the solution, resulting in a significant increase in the electrochemical activity of the system. In the case of the solution with added glycine, a significant increase in cathodic currents and the appearance of an anodic peak at a current density exceeding 300 mA/cm^2^ at a potential of approximately −0.1 V were observed, which is evidence of samarium co-deposition and the formation of a Co-Sm alloy phase. The system with L-arginine exhibited a different pattern, with inhibition of electrode processes observed. Despite the presence of Sm(III) ions, the current densities remained significantly lower than in the system with glycine, and the oxidation peak shifted toward lower potentials and became flattened. This suggests that L-arginine, through the formation of stable complexes or strong adsorption on the cathode surface, increases the energy barrier for reduction and hinders solid phase nucleation.

### 3.1. L-Arginine as a Complexing Agent

**Figure 3:**
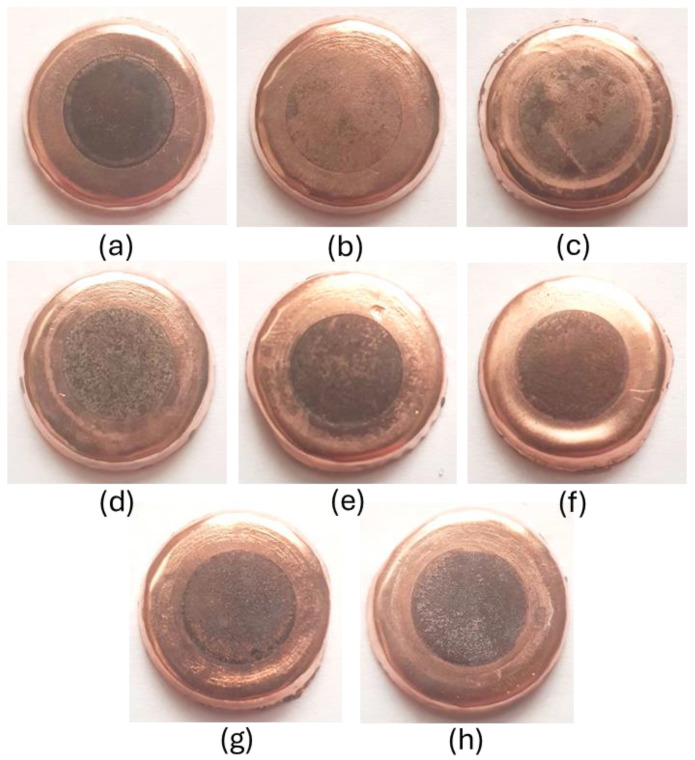
Samples deposited from solutions containing L-arginine at a potential of (**a**) −1.1 V; (**b**) −1.2 V; (**c**) −1.3 V; (**d**) −1.4 V; (**e**) −1.5 V; (**f**) −1.6 V; (**g**) −1.7 V; (**h**) −1.8 V.

The coatings were deposited from electrolyte I (Table 1) containing 0.05 M Sm_2_O_3_, 0.1 M CoCl_2_·6H_2_O, and 0.2 M L-arginine. The layers were deposited at pH = 3.2 with an electrodeposition time of 30 min. A fresh portion of the solution was used for each deposition. Each layer was deposited and analyzed on the same day. The photos below present (Figure 3) the effect of the applied potential on the appearance of the obtained coatings:

Visual analysis of samples obtained by electrodeposition of an Sm-Co alloy on a copper substrate demonstrates a correlation between the applied potential and the quality of the deposit. At a potential of −1.1 V (Figure 3a), the deposit forms a dark, almost black, and matte coating with a high degree of uniformity. At a potential of −1.2 V (Figure 3b), where the working area is almost completely devoid of visible deposit, the sample deposited at a potential of −1.3 V (Figure 3c) is characterized by a small amount of deposit, which is unstable. Further reduction in the potential from −1.4 V to −1.6 V (Figure 3d–f) leads to a gradual darkening of the deposit again. At the lowest potentials, i.e., −1.7 V (Figure 3g) and −1.8 V (Figure 3h), the deposit becomes dark gray. The degradation of the coatings is a direct result of the intense co-evolution of hydrogen. Hydrogen gas, released on the cathode surface, disrupts the metal growth process, leading to the formation of inhomogeneous structures and limited adhesion to the copper substrate.

**Figure 4:**
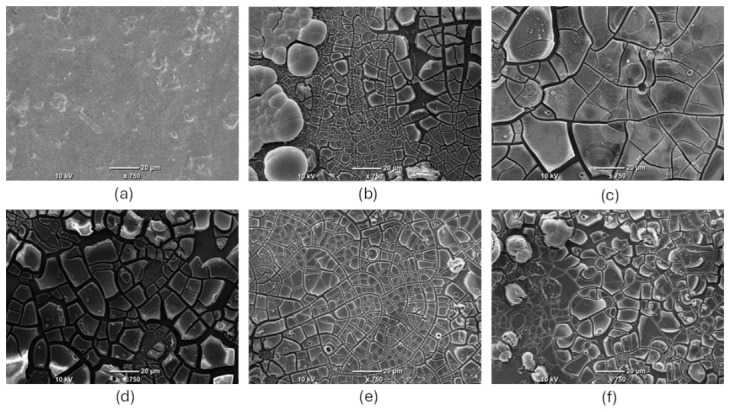
SEM analysis of the Sm-Co layer with L-arginine as a complexing agent at 750× magnification (**a**) −1.1 V; (**b**) −1.4 V; (**c**) −1.5 V; (**d**) −1.6 V; (**e**) −1.7 V; (**f**) −1.8 V.

The effect of the applied potential on the morphology of Sm-Co alloy coatings deposited from a solution containing L-arginine as a complexing agent was investigated (Figure 4). Due to the lack of stability of the obtained layers at the potential of −1.2 V and −1.3 V, these samples were not subjected to SEM analysis:

Observation of the sample obtained at the lowest potential of −1.1 V (Figure 4a) shows the formation of a continuous and highly homogeneous layer. No surface defects or cracks were observed at this stage of deposition. As the potential shifts towards more negative values, i.e., −1.4 V (Figure 4b), a clear change in the growth mechanism occurs—the first spherical agglomerates appear, along with a network of cracks, signaling an increase in internal stresses in the deposit. Coatings obtained at potentials of −1.5 V (Figure 4c) and −1.6 V (Figure 4d) are characterized by large grains. Sample (Figure 4d) has the largest intergrain gaps of all the samples tested. This phenomenon results from high tensile stresses, most likely generated by intense hydrogen co-evolution. Further reduction in the potential to −1.7 V (Figure 4e) and −1.8 V (Figure 4f) leads to progressive fragmentation. At a potential of −1.7 V, the crack network becomes significantly denser. In turn, at a value of −1.8 V (Figure 4f), a morphology is observed in which lump-shaped forms appear, built up on a strongly cracked matrix.

**Figure 5:**
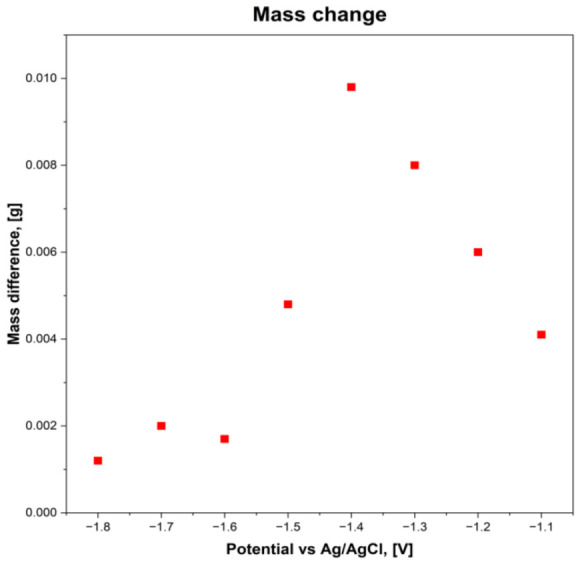
Mass of the deposited coating with the usage of L-arginine as a complexing agent depending on the variable potential. The red squares represent experimental data points.

The next stage of the research was the analysis of the change in the mass of the deposited alloy depending on the change in potential, as shown in Figure 5.

Analysis of the obtained data indicates a nonlinear mass increase with a clearly marked extreme. At the initial potential of −1.1 V, the mass increase in the deposited material is 0.004 g. Lowering the potential to −1.2 V causes the mass of the deposited layer to increase to 0.006 g. When the potential is reduced to −1.3 V, the deposit has a mass of 0.008 g. The maximum value, close to 0.01 g, was observed at a potential of −1.4 V, which under the studied conditions is the optimal point for the co-deposition kinetics of samarium and cobalt. A further shift in the potential towards more negative values results in a decrease in the mass of the deposit. At a potential of −1.5 V, it is 0.0048 g. At a potential of −1.6 V, the mass of the deposit decreases to 0.0017 g, and in the range from −1.7 V to −1.8 V, it stabilizes at minimum values oscillating around 0.0016 g.

**Figure 6:**
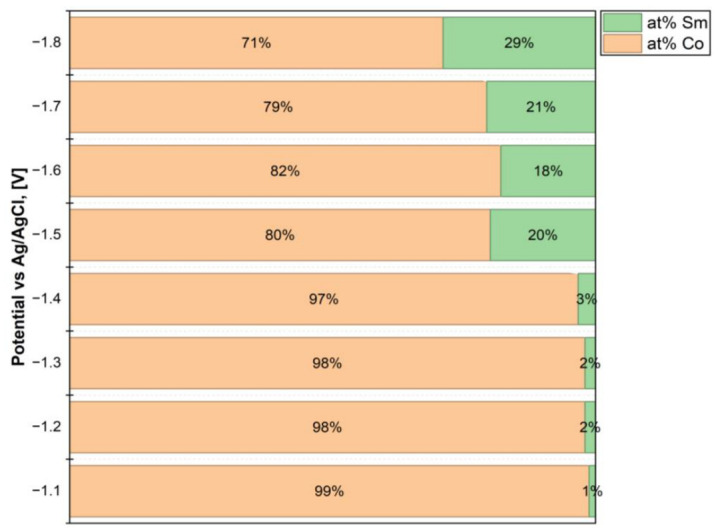
Composition of deposited alloy with L-arginine as a complexing agent [at.%].

The next step was to determine the content of individual elements in the deposited alloy by determining the relationship between cobalt and samarium content as a function of the applied potential using XRF. The results presented in Figure 6 reveal the dependence of the alloy composition on the process parameters.

In the potential range from −1.1 V to −1.4 V vs. Ag/AgCl, the process results in a small amount of samarium obtained in the deposit. At a potential of −1.1 V, the samarium content is low, at approximately 1 at.%. Shifting the potential to −1.2 V and −1.3 V results in a slight increase in the Sm content, to 2 at.% at both potentials. At a potential of −1.4 V, the samarium content remains low, reaching approximately 3 at.%. This situation changes after exceeding the potential of −1.4 V, which is reflected in a significant jump in samarium content on the graph. At an applied potential of −1.5 V, the Sm content increases to 20 at.%. At the next measurement point, at a potential of −1.6 V, a Sm content of approximately 18 at.% is observed. Lowering the potential to −1.7 V again shifts the equilibrium towards samarium, increasing its share to 21 at.%. At the most negative potential tested, −1.8 V, it can be seen that the samarium content reached its maximum value of approximately 29 at.%.

**Figure 7:**
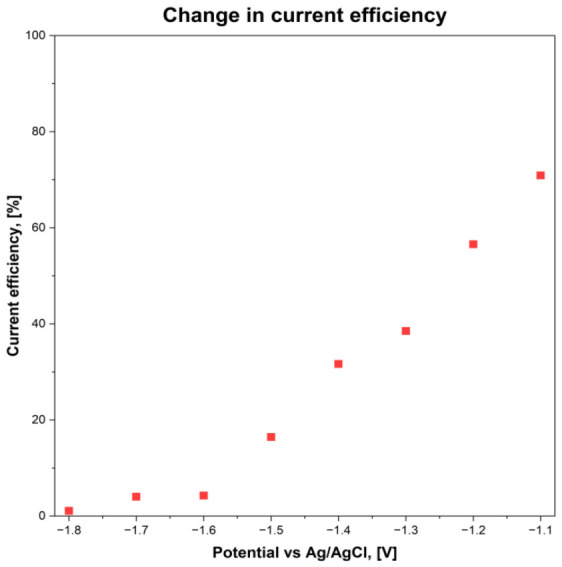
Change in current efficiency depending on the variable potential for deposition from a solution containing L-arginine. The red squares represent experimental data points.

Based on the obtained Sm and Co contents from Figure 6 and Equations (1) and (2), the current efficiency was determined depending on the applied potential (Figure 7).

The highest current efficiency of 71% occur at −1.1 V. As the potential is reduced, the current efficiency begins to decrease. At a potential of −1.2 V, it drops to 57%. The current efficiency is almost 39% for the sample deposited at a potential of −1.3 V. Further potential reduction leads to an efficiency of 32% at −1.4 V. Reducing the potential to −1.5 V yields a value of 16%. At potentials of −1.6 V and −1.7 V, the current efficiency stabilizes at 4%. However, further potential reduction also leads to a decrease to 2%. This phenomenon confirms the dominance of the hydrogen evolution reaction at high potentials.

**Figure 8:**
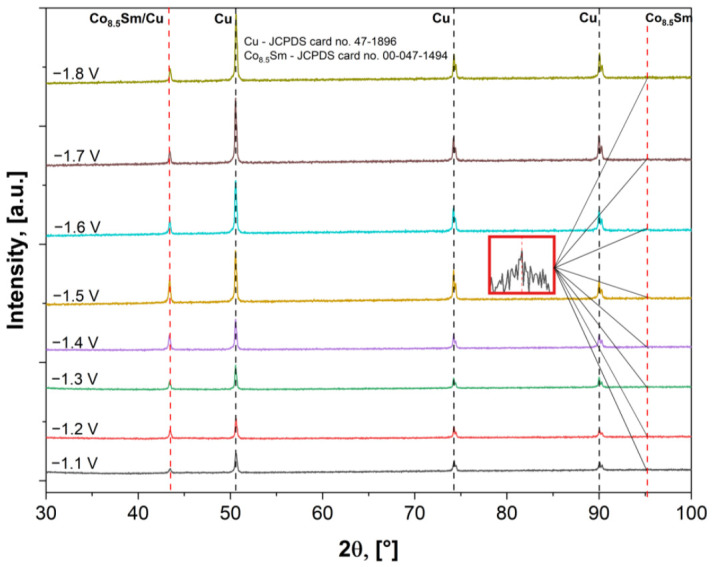
XRD analysis for the layer electrodeposited with L-arginine as a complexing agent.

To determine the influence of electrodeposition parameters on the crystal structure and phase composition, XRD structural analysis was performed (Figure 8).

All recorded diffraction patterns are dominated by intense reflections at angles of 44°, 50°, 74°, and 90°, and a faint peak at 95°. At angles of 44° and 95°, the peaks appearing were identified as the signal originating from copper and the Co_8.5_Sm intermetallic phase (JCPDS card No. 00-047-1494). At angles of 50°, 74°, and 90°, the peaks appearing were assigned to the (1 1 1) and (2 0 0) crystallographic planes of face-centered cubic copper, which is the substrate (JCPDS card No. 47-1896). At an angle of 44°, the peaks originating from the Co_8.5_Sm intermetallic phase and copper overlap. However, the peak at 94° related only to the cobalt–samarium phase confirmed the presence of alloying alloy on the surface. The height of the peak is low intensity due to the thickness of the deposit. To evaluate the crystalline structure of the deposited material, the experimental 2θ angles and d-spacing were compared with the reference data in Table 2.

The positions of the recorded diffraction peaks show a very good agreement with the reference values from JCPDS card No. 00-047-1494, confirming the successful synthesis of the target phase at a potential of −1.5 V. This comparison enables reliable structural verification and confirms the Co_8.5_Sm phase in the analyzed sample. Discrepancies may result from the use of a different tube than the one specified in the JCPDS card.

For a more detailed analysis of the chemical composition, EDS analysis was performed, the results of which are presented in Table 3:

We can distinguish three sources of carbon: carbon dioxide absorbed from in the air, carbon from the added complexing agent used, and impurities resulting from sample transfer. For the highest carbon content, i.e., 41 at.% at a potential of −1.1 V, samarium was not identified. At potentials of −1.2 V and −1.3 V, EDS analysis could not be performed due to the lack of stability of the deposited layer. As the carbon amount decreases, the samarium content is at a level of 10–12 at.% at various deposition potentials. At a potential of −1.5 V, the carbon content increases to 23 at.%, and the samarium content decreases to 8 at.%. The higher the percentage of carbon, the more samarium deposition is inhibited.

To investigate the form in which the elements occur on the surface, XPS analysis was performed for the sample obtained at a deposition potential of −1.5 V vs. Ag/AgCl (Table 4):

Based on the XPS results, cobalt and samarium were detected in the form of hydroxides and oxides. The cobalt ion content was 13.7 at.%, and the samarium ion content was 6 at.%. These elements were not detected in metallic form.

### 3.2. Glycine as a Complexing Agent

**Figure 9:**
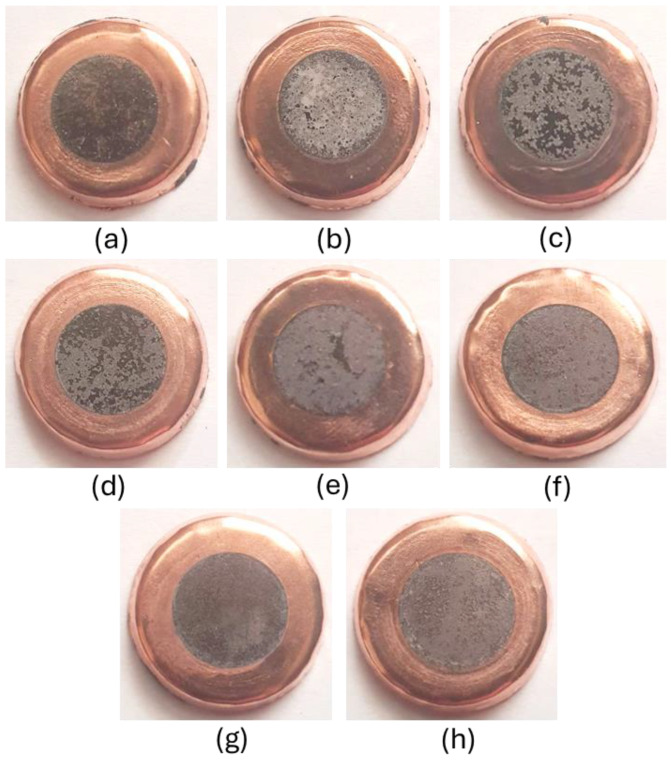
Samples deposited from solutions containing glycine at a potential of (**a**) −1.1 V; (**b**) −1.2 V; (**c**) −1.3 V; (**d**) −1.4 V; (**e**) −1.5 V; (**f**) −1.6 V; (**g**) −1.7 V; (**h**) −1.8 V.

In the next experiment, the complexing agent used was changed. This time, glycine was used as a substitute for arginine. The coatings were deposited from electrolyte II (Table 1) containing 0.05 M Sm_2_O_3_, 0.1 M CoCl_2_·6H_2_O, and 0.2 M glycine. The layers were deposited at pH = 3.2 with an electrodeposition time of 30 min. A fresh portion of the solution was used for each deposition test. Each layer was deposited and analyzed on the same day. The photos show the effect of the applied potential on the appearance of the obtained coatings (Figure 9).

The sample obtained at a potential of −1.1 V (Figure 9a) is characterized by a high degree of continuity and a dark color. The situation changes in the potential range from −1.2 V to −1.4 V, corresponding to samples (Figure 9b–d), where a clear change in character is observed. At a potential of −1.2 V (Figure 9b), the gray deposit dominates. At potentials of −1.3 V (Figure 9c) and −1.4 V (Figure 9d), an increase in the proportion of dark structures is observed. For the layer deposited at −1.5 V (Figure 9e), the gray part of the deposit dominates, with a small proportion of dark agglomerates. Further reduction in the potential leads to re-homogeneity of the deposit, as illustrated by samples −1.5 V (Figure 9e) and −1.6 V (Figure 9f), on which the surface is uniform and covers the copper substrate. The surface of the sample obtained at a potential of −1.6 V (Figure 9f) already presents a uniform, gray layer. The layers documented in the photos −1.7 V (Figure 9g) and −1.8 V (Figure 9h) take on a light gray color.

**Figure 10:**
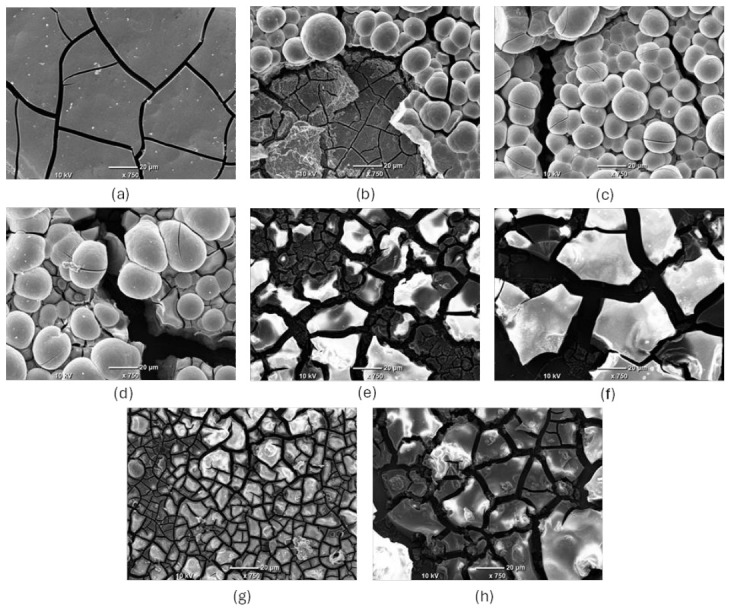
SEM analysis of the Sm-Co layer with glycine as a complexing agent at 750× magnification (**a**) −1.1 V; (**b**) −1.2 V; (**c**) −1.3 V; (**d**) −1.4 V; (**e**) −1.5 V; (**f**) −1.6 V; (**g**) −1.7 V; (**h**) −1.8 V.

SEM analysis was performed to investigate the effect of applied potential, along with glycine as a complexing agent, on the morphology of the solution-deposited coatings (Figure 10).

In the region of the most positive potentials, represented by the −1.1 V sample (Figure 10a), the deposit is characterized by the greatest homogeneity. Transitioning to a potential of −1.2 V (Figure 10b) causes the crack network to deepen, and spherical structures begin to appear, building up on the cracked layer. At a potential of −1.3 V (Figure 10c), spherical agglomerates begin to dominate. Further potential reduction leads to an increase in the number of cracks. For the −1.4 V sample (Figure 10d), deep and wide cracks are visible. At a potential of −1.5 V (Figure 10e), intensification of surface fragmentation and the disappearance of spherical agglomerates can be observed. This fragmentation is a direct result of intense hydrogen co-evolution. The sample obtained at a potential of −1.6 V (Figure 10f) is characterized by the least surface fragmentation and the largest cracks. Further reduction in the potential to −1.7 V (Figure 10g) leads to progressive defragmentation of the surface of the obtained layer. At a potential of −1.8 V (Figure 10h), grains with the greatest size difference occur.

Comparative analysis with the L-arginine-containing system reveals discrepancies in the morphological evolution of the Sm-Co precipitates due to the choice of complexing agent. Comparison of the morphology of the Sm-Co layers indicates that the type of amino acid used determines the precipitate growth mechanism: the presence of arginine (Figure 4) favors the formation of flat layers with a regular network of cracks, while the use of glycine (Figure 10) leads to structures with a greater tendency to spherical and agglomerative growth.

**Figure 11:**
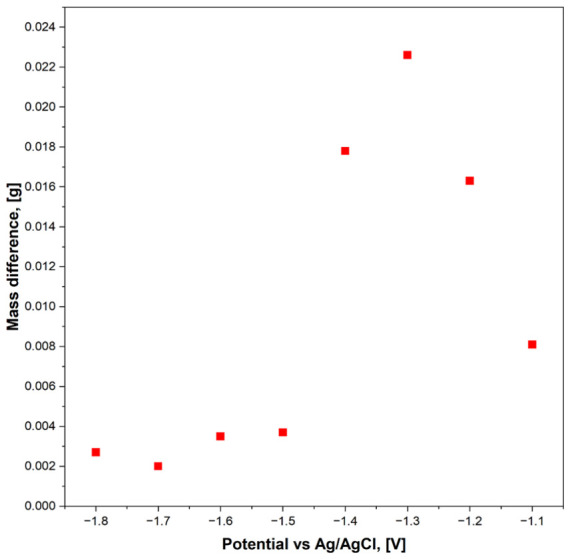
Mass of the deposited coating with the usage of glycine as a complexing agent depending on the variable potential. The red squares represent experimental data points.

After morphological analysis of the deposit, the changes in the mass of the deposited alloy were analyzed as a function of the potential change. The results are presented in Figure 11.

At a potential of −1.1 V, the precipitate mass is 0.008 g, and as the potential is shifted to −1.2 V, it doubles to 0.016 g. At a potential of −1.3 V, the highest mass difference of 0.0225 g is noted. After crossing the −1.3 V threshold, a break in the curve is visible. At a potential of −1.4 V, the mass drops to 0.018 g, and at −1.5 V, the deposit amount decreases significantly to 0.0038 g. In the further potential range, from −1.6 V to −1.8 V, the precipitate mass stabilizes at a very low, almost constant level, oscillating between 0.002 g and 0.0035 g. The next step was to determine the percentage content of individual elements using XRF analysis.

**Figure 12:**
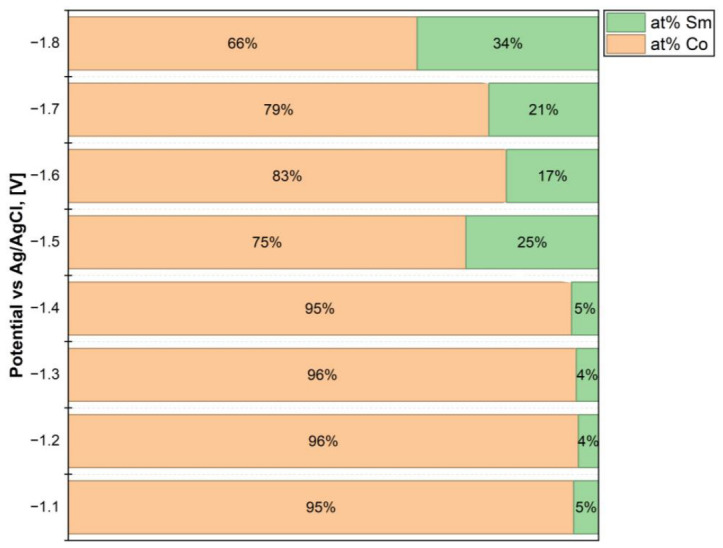
Composition of deposited alloy with glycine as a complexing agent [at.%].

The results obtained by XRF, summarized in Figure 12, reveal the existence of distinct potential thresholds that determine the efficiency of samarium co-deposition.

Within the tested potential range, spanning from −1.1 V to −1.4 V vs. Ag/AgCl, the deposition process is characterized by a dominance of cobalt with a low samarium content. At a potential of −1.1 V, the samarium content is approximately 5 at.%. Lowering the potential to −1.2 V and −1.3 V leads to minor composition changes, with the Sm content reaching 4 at%. At a potential of −1.4 V, the samarium content increases to approximately 5 at.%. A breakthrough in the layer formation mechanism becomes visible after crossing the −1.4 V barrier, as evidenced on the graph by a distinct jump in samarium content. At an applied potential of −1.5 V, the Sm content increases nearly fivefold compared to the previous potential, reaching a value of approximately 25 at.%. In the next measurements, for a potential of −1.6 V, the samarium content is recorded at about 17 at.%, and at −1.7 V, it reaches about 21 at.%. The largest change in chemical composition was recorded at the most negative potential tested −1.8 V, where the samarium content increases to a maximum of about 34 at.%.

The use of glycine leads to a higher samarium content at low potentials compared to the system with L-arginine. At a potential of −1.1 V, the samarium content in the deposit obtained from the glycine bath is approximately 5 at.%, while in the presence of arginine, it is lower at approximately 1 at.%. At a potential of −1.4 V, the Sm content for both complexing agents remains at a low level, 5 at.% for glycine vs. 3 at.% for arginine. The difference is noted in the region of the most negative potentials from −1.5 V to −1.8 V, where L-arginine enables more effective induced co-deposition in relation to the overall mass, though in atomic terms, glycine still results in a high samarium fraction of 34 at.% at the lowest potential.

**Figure 13:**
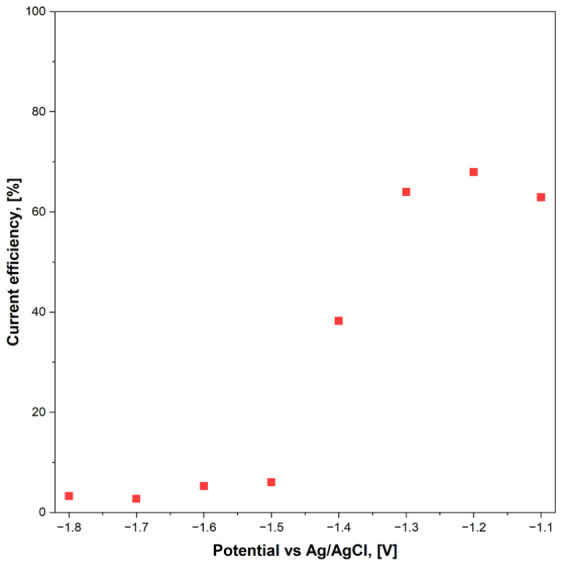
Change in current efficiency depending on the variable potential for deposition from a solution containing glycine. The red squares represent experimental data points.

The study of the electrodeposition process of the Sm-Co alloy on a copper substrate allowed the determination of the relationship between the applied cathodic potential and the current efficiency of the deposition, shown in Figure 13.

Analysis of the curve (Figure 13) reveals a clear increase in efficiency in the potential range from −1.1 V to −1.3 V vs. Ag/AgCl. At the most positive potential, i.e., −1.1 V, the current efficiency is 63%. Lowering the potential to −1.2 V increases it to a maximum value of 68%. Further potential reductions lead to a decrease in current efficiency. At a potential of −1.3 V, it oscillates at 64%. At a potential of −1.4 V, there is a significant drop in efficiency to 38%. Moving to a potential of −1.5 V leads to another significant drop in efficiency, this time to 6%. At increasingly negative potentials, the current efficiency stabilizes at a few percent. Applying a potential of −1.6 V leads to an efficiency of 5%. Samples obtained at the most negative potentials, i.e., −1.7 V and −1.8 V, are characterized by an efficiency of approximately 3%.

Analysis of the current efficiency of the process reveals differences in the stability of the two electrolytic systems. In the system containing glycine, the highest efficiency of 68% was observed at a potential of −1.2 V, but further shifting the potential towards more negative values results in a drop in efficiency to 6% at −1.5 V. In contrast, the bath with added L-arginine is characterized by a wider window of process stability; the efficiency at −1.1 V is similar (approximately 70%). At potentials of −1.2 V and −1.3 V, electrodeposition from the glycine-containing solution demonstrates increased current efficiency compared to arginine. In the potential range of −1.4 V to −1.5 V, the current efficiency for the glycine solution is lower than for the arginine solution. Subsequent samples obtained at lower potentials, i.e., from −1.6 V to −1.8 V, exhibit similar current efficiencies for both solutions, at the level of a few percent. While glycine favors deposition kinetics only in a narrow energy range, L-arginine provides the stability necessary for the controlled co-deposition of samarium and cobalt under strong polarization conditions.

**Figure 14:**
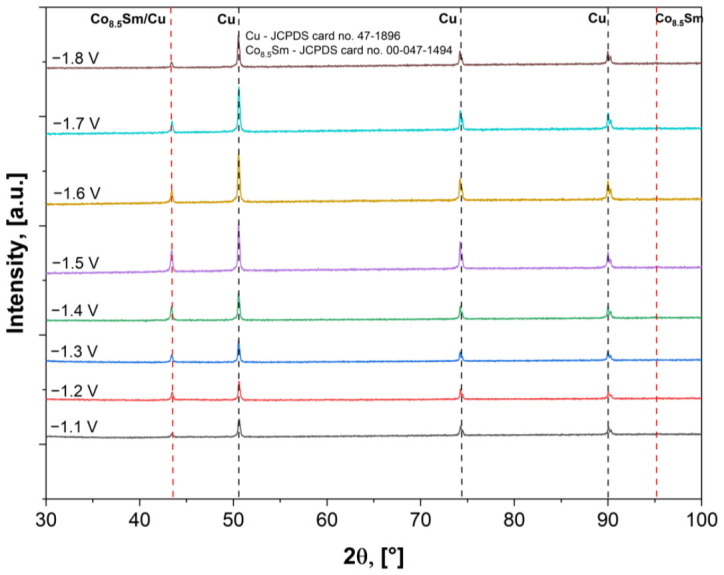
XRD analysis for the layer electrodeposited with glycine as a complexing agent.

To determine the influence of electrodeposition parameters on the crystal structure and phase composition, a diffraction structural analysis was performed (Figure 14):

All recorded diffractions are characterized by intense reflections at angles of 44°, 50°, 74°, and 90°, with an additional, weaker peak visible near 95°. The peaks at angles of 44° and 95° were identified as a signal originating from copper and the Co_8.5_Sm intermetallic phase (JCPDS card No. 00-047-1494). Reflections at angles of 50°, 74°, and 90° were assigned to crystallographic planes of face-centered cubic copper, which constitutes the substrate (JCPDS card No. 47-1896). At an angle of approximately 44°, the peaks originating from the Co_8.5_Sm intermetallic phase and the copper substrate overlap. The XRD results are analogous to those obtained for the L-arginine system. Regardless of the applied potential and the complexing agent used, the peaks appear at the same angles.

To analyze the chemical composition, EDS analysis was performed, the results of which are presented in Table 5.

Carbon sources are the same as in the previous case. At the most positive potentials, i.e., from −1.1 V to −1.4 V, the samarium content remains at 3–5 at.%. Between the potentials of −1.4 V and −1.5 V, there is a significant increase in samarium content to 11 at.%. At potentials lower than −1.5 V, the chemical composition stabilizes, and the percentages of individual elements remain similar. In the case of deposition from a glycine solution, there is no visible correlation between the carbon content in the alloy and the samarium content in the resulting layer.

To investigate the form in which the elements occur on the surface, XPS analysis was performed for the sample obtained at a deposition potential of −1.5 V vs. Ag/AgCl (Table 6):

Based on the XPS results, cobalt and samarium were found to occur in the form of hydroxides and oxides. The cobalt ion content was 8.6 at.%, and the samarium ion content was 7.8 at.%. These elements were not found in metallic form.

L-arginine demonstrates a more favorable effect on the stability of the electrodeposition process and a higher morphological quality of the resulting Sm-Co coatings compared to the glycine system. A key argument for choosing arginine was the ability to stably conduct the induced co-deposition process at low cathodic potentials, which allowed for obtaining a nearly equimolar ratio of elements and a stable Co_8.5_Sm intermetallic phase.

### 3.3. Influence of the Aging Time of the L-Arginine Solution on the Electrodeposition Process

**Figure 15:**
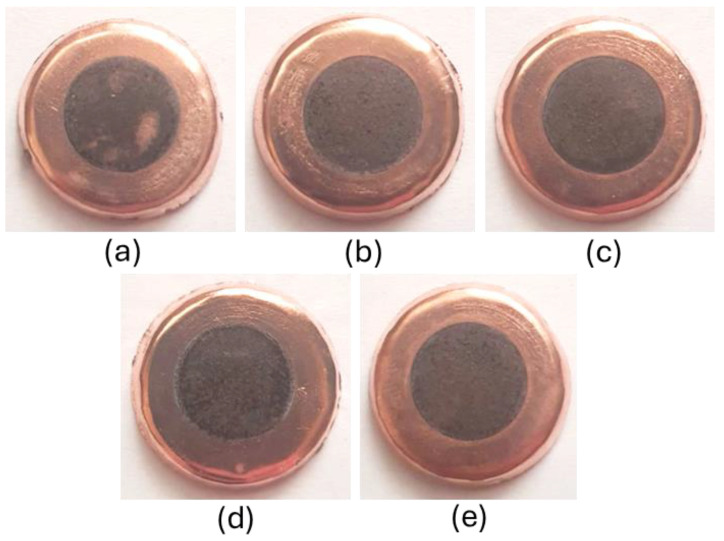
Samples after electrodeposition from L-arginine-containing solution aged for (**a**) 1 day; (**b**) 2 days; (**c**) 3 days; (**d**) 4 days; (**e**) 7 days.

In the next experiment, the effect of solution aging times of 1, 2, 3, 4, and 7 days on the electrodeposition process with arginine as the complexing agent was investigated. The solution was unstirred. The coatings were deposited from electrolyte I (Table 1) containing 0.05 M Sm_2_O_3_, 0.1 M CoCl_2_·6H_2_O, and 0.2 M arginine. The layers were deposited at pH = 3.2 with an electrodeposition time of 30 min. A fresh portion of the solution was used for each deposition test. Layers were deposited after 1, 2, 3, 4, and 7 days of solution aging. The deposit was analyzed on the same day as the deposition. The deposition potential was −1.5 V vs. Ag/AgCl. The following photos show the effect of solution aging on the appearance of the obtained coatings (Figure 15):

The presented photographs show the effect of aging time on the appearance of the deposited coatings after aging the solution for 1, 2, 3, 4, and 7 days. Sample (Figure 15a) obtained from a solution aged for 1 day, shows a non-uniformly colored deposit with clear traces of copper substrate. With electrolyte aging, as documented in samples (Figure 15b) (2 days) and (Figure 15c) (3 days), the deposit’s color becomes more uniform. The surface becomes darker and more uniform. Sample (Figure 15b) still exhibits small, almost invisible, darker, spotty differences in texture. In photo (Figure 15c), a uniform dark deposit can be observed, although at one point the layer is clearly thinner, as a trace of copper begins to become visible. The sample obtained from the solution aged for 4 days (Figure 15d) is characterized by a gray texture in its central part. Darker clusters appear at the edges of the deposited layer. In the last photo (Figure 15e) the sediment after 7 days shows a similar degree of homogeneity as sample (Figure 15b).

**Figure 16:**
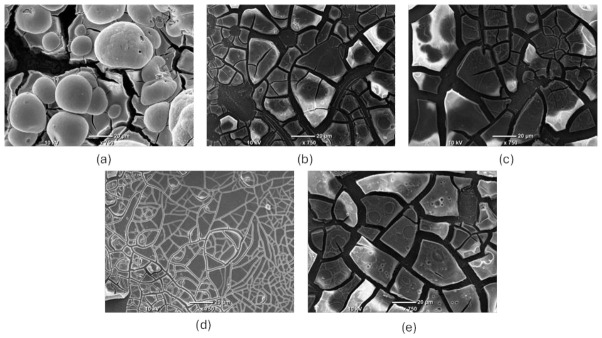
SEM analysis of the Sm-Co layer from the solution aged with L-arginine as a complexing agent at 750× magnification: (**a**) 1 day; (**b**) 2 days; (**c**) 3 days; (**d**) 4 days; (**e**) 7 days.

To further investigate the obtained coatings with aged solutions, SEM analysis was performed (Figure 16):

On the first day after bath preparation, as shown in sample (Figure 16a)—1 day, spherical structures predominate. Deep crevices are visible between individual agglomerates, suggesting high internal stresses. The morphological change in the deposited layer occurs after 2 days (Figure 16b) of solution aging, where the formation of flat structures replaces spherical growth. The deposit obtained from the solution aged for 3 days (Figure 16c) looks the same as that obtained from the solution aged for 2 days. In both cases, numerous cracks and crevices are visible. In the solution aged for 4 days, the most intense fragmentation of the deposited layer can be observed. Numerous shallow cracks predominate. The resulting grains are the smallest. The structural changes are documented in a photograph taken after 7 days (Figure 16e) of bath aging. The coating begins to adopt the structure seen in samples (Figure 16b) and (Figure 16c). Large grains with deep crevices between them dominate.

**Figure 17:**
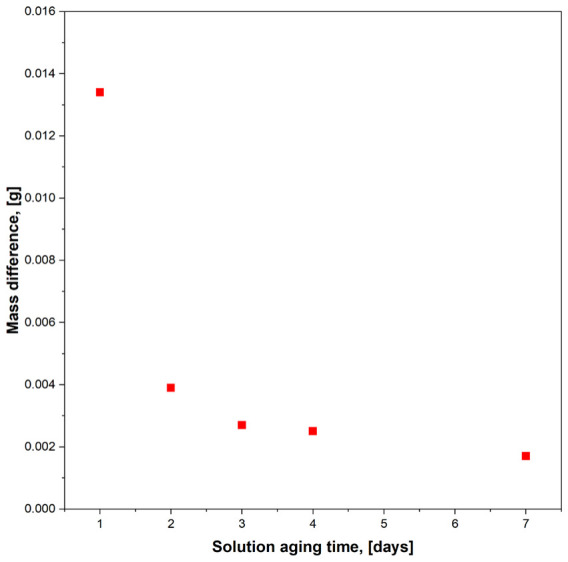
Mass of the deposited coating with the usage of L-arginine as a complexing agent depending on the solution aging time. The red squares represent experimental data points.

After surface analysis, the change in the mass of the deposited alloy was determined depending on the solution aging time (Figure 17).

The study of the effect of solution aging time on the mass of the deposited alloy, presented in Figure 17, reveals that the system exhibits its highest efficiency after the first day after electrolyte preparation, reaching a deposit mass of approximately 0.0134 g. After the second day of aging, there is a clear decrease in the mass of the deposited layer to the level of 0.0039 g. In the subsequent stages, including the third and fourth day of solution aging, the mass differences stabilize to 0.0027 g and 0.0025 g, respectively. In contrast to the beginning of the process, the final section of the curve between the fourth and seventh day shows a slight decrease, leading to a deposit mass of approximately 0.0017 g on the seventh day.

**Figure 18:**
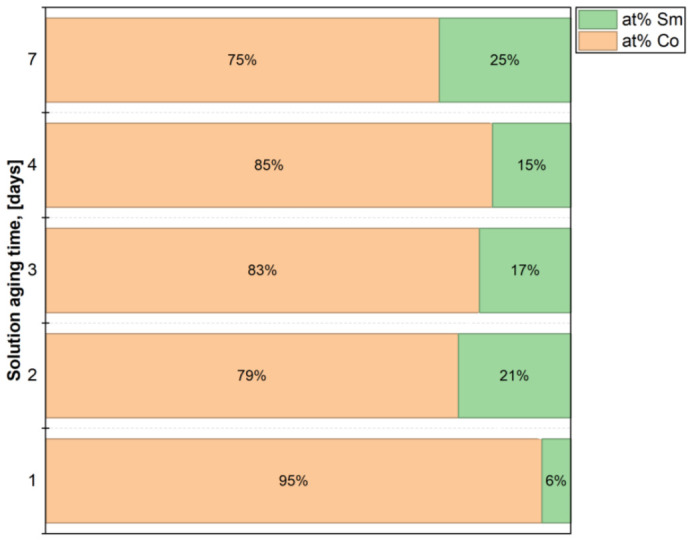
Composition of the alloy deposited from the solution aged with L-arginine as a complexing agent [at.%].

Electrolyte aging is crucial for the repeatability of the electrodeposition process on both laboratory and industrial scales. The chemical composition analysis results, summarized in the graph, illustrate the change in cobalt and samarium content in the deposits obtained from solutions aged for 1, 2, 3, 4, and 7 days (Figure 18):

On the first day after solution preparation, the samarium content is approximately 6 at.%. This situation changes after two days of aging, when the samarium content increases to approximately 21 at.%, representing a nearly fourfold increase in its atomic presence in the alloy compared to day 1. On the third day of solution aging, a change in composition is observed, with the samarium content stabilizing at approximately 17 at.%. On the fourth day, its concentration in the layer decreases again to approximately 15 at.%. Analysis conducted after seven days of electrolyte aging indicates a further increase in samarium deposition efficiency, with its share in the layer reaching the maximum recorded value of approximately 25 at.%. The final data set shows that the samarium content in the deposit fluctuates over the period studied—from 6 at.% to 25 at.%—demonstrating that electrolyte age is a critical parameter influencing the reaction kinetics and the resulting chemical composition of the deposited layer.

**Figure 19:**
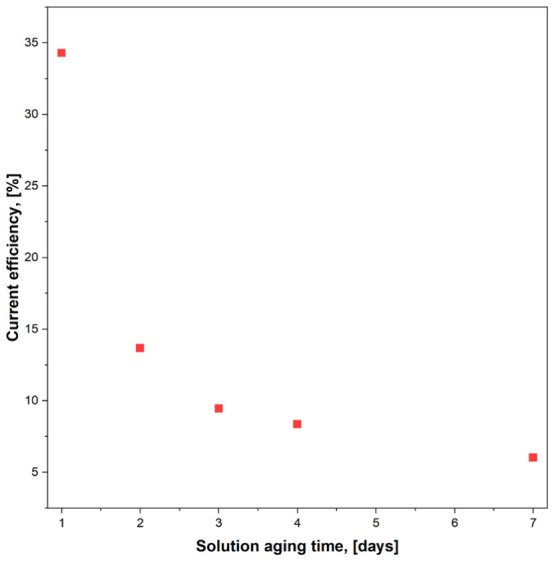
Change in current efficiency depending on the solution aging time for deposition from a solution containing L-arginine. The red squares represent experimental data points.

An important step in assessing the operational stability of the tested electrochemical system was to determine the effect of the solution aging time on its ability to effectively transfer charge, which was illustrated in the graph of the change in current efficiency as a function of aging time (Figure 19).

On the first day after solution preparation, the system exhibits peak performance, reaching a current efficiency of approximately 34%. Already after the second day of aging, a decline in performance was observed, dropping to approximately 14%. For solutions aged for 3, 4, and 7 days, the degradation process slows down, and the current efficiency curve undergoes a slight, linear decline. Between the third and seventh day, the current efficiency decreases from approximately 10% to just 6%, where it reaches its minimum.

**Figure 20:**
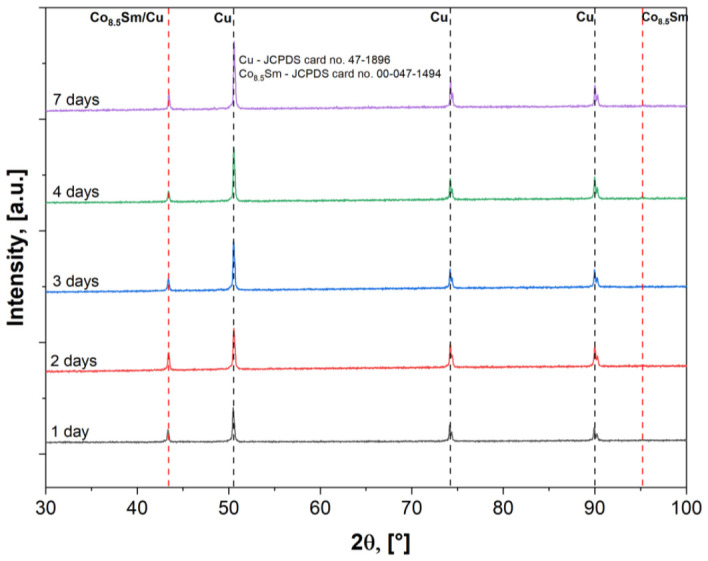
XRD analysis of the electrodeposited layer from the aged solution with L-arginine as a complexing agent.

The stability studies of the system are complemented by an assessment of the effect of electrolyte storage time on the structural quality of the resulting coatings. Below is a diffraction analysis of a series of samples obtained from the aging solution (Figure 20).

All recorded diffraction patterns are consistent with the results obtained for freshly prepared solutions and are dominated by intense reflections at angles of 44°, 50°, 74°, and 90°, and a faint peak at 95°. Regardless of the electrolyte aging time, the peaks consistently appear at the same diffraction angles. At angles of 44° and 95°, the reflections were identified as the signal originating from copper and the Co_8.5_Sm intermetallic phase (JCPDS card No. 00-047-1494). The peaks appearing at angles of 50°, 74°, and 90° were assigned to the crystallographic planes of face-centered cubic copper, which constitutes the substrate (JCPDS card No. 47-1896).

### 3.4. Influence of the Aging Time of the Glycine Solution on the Electrodeposition Process

**Figure 21:**
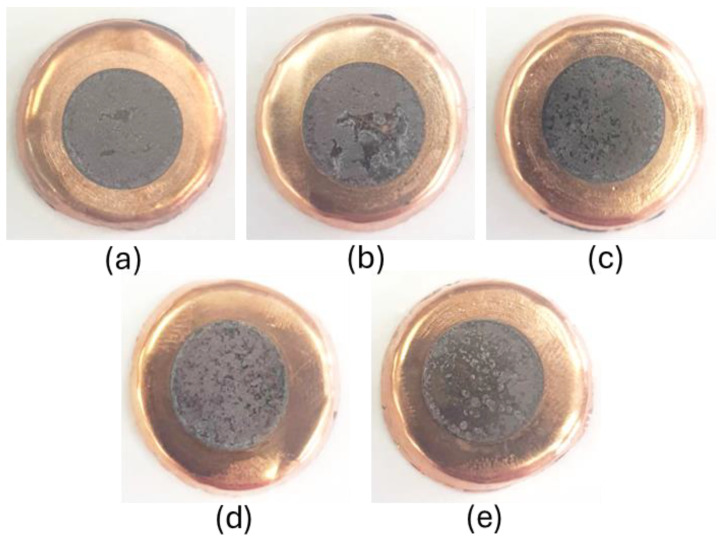
Samples after electrodeposition from glycine-containing solution aged for (**a**) 1 day; (**b**) 2 days; (**c**) 3 days; (**d**) 4 days; (**e**) 7 days.

In the next part of the experiment, the effect of solution aging times of 1, 2, 3, 4, and 7 days on the electrodeposition process was again examined, this time with glycine as the complexing agent. The solution was unstirred. The coatings were deposited from an electrolyte II (Table 1) containing 0.05 M Sm_2_O_3_, 0.1 M CoCl_2_·6H_2_O, and 0.2 M glycine. The layers were deposited at pH = 3.2 with an electrodeposition time of 30 min. A fresh portion of the solution was used for each deposition test. Layers were deposited after 1, 2, 3, 4, and 7 days of solution aging. The deposit was analyzed on the same day as the deposition. The deposition potential was −1.5 V vs. Ag/AgCl. The photos below show the effect of increased samarium content and applied potential on the appearance of the obtained coatings (Figure 21):

With the electrolyte aging, the 1-day sample (Figure 21a) shows a uniform, dark deposit. Over time, minimal changes in the surface structure are observed. Sample (Figure 21b) (2 days of electrolyte aging) shows localized traces of the deposit’s texture, originating from the copper substrate. In photos (3 days), (Figure 21c) and (Figure 21d) (4 days), the layer becomes completely uniform and dark again. Samples obtained from the aged solution are characterized by uniform color and texture. In the last photo (Figure 21e), the deposit after 7 days shows a slightly more varied texture with visible lighter traces. This sample is characterized by the least uniformity of the obtained layer compared to the previous samples.

**Figure 22:**
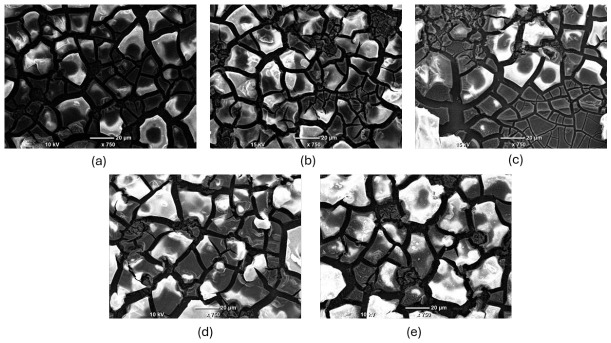
SEM analysis of the Sm-Co layer from the solution aged with glycine as a complexing agent at 750× magnification: (**a**) 1 day; (**b**) 2 days; (**c**) 3 days; (**d**) 4 days; (**e**) 7 days.

To further determine the morphology of the coatings obtained with the aged solutions, SEM analysis was performed (Figure 22):

Figure 22 shows the effect of bath aging time on the morphology of the Sm-Co layer. Sample (Figure 22a) (1 day) shows flat structures with wide crevices. As time passes, as shown in samples (Figure 22b) (2 days) and (Figure 22c) (3 days), the precipitate fragments and the crack network become denser. The sample obtained after 4 days (Figure 22d) is characterized by the formation of slightly larger structures while maintaining a high degree of cracking. In the last photo (Figure 22e), the precipitate after 7 days shows a stable structure with distinct crevices, similar to sample (Figure 22d).

**Figure 23:**
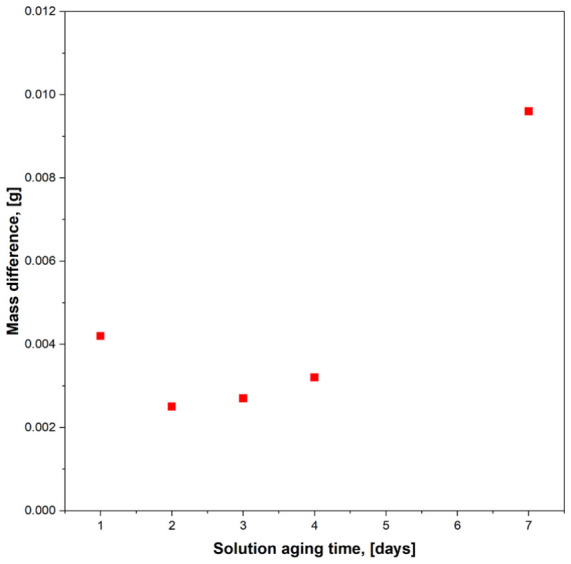
Mass of the deposited coating with the usage of glycine as a complexing agent depending on the solution aging time. The red squares represent experimental data points.

After surface analysis, the change in the mass of the deposited alloy was determined depending on the solution aging time (Figure 23):

The mass analysis of the deposit after the first day is approximately 0.0042 g. On the second day of aging, it decreases to the lowest level of 0.0025 g. In the next stages (3rd and 4th day), a slight increase in mass is observed, to 0.0027 g and 0.0032 g, respectively. Unlike in the case of the aged solution with L-arginine, the final stage of aging is characterized by a significant increase in mass, reaching the highest value of approximately 0.0096 g.

**Figure 24:**
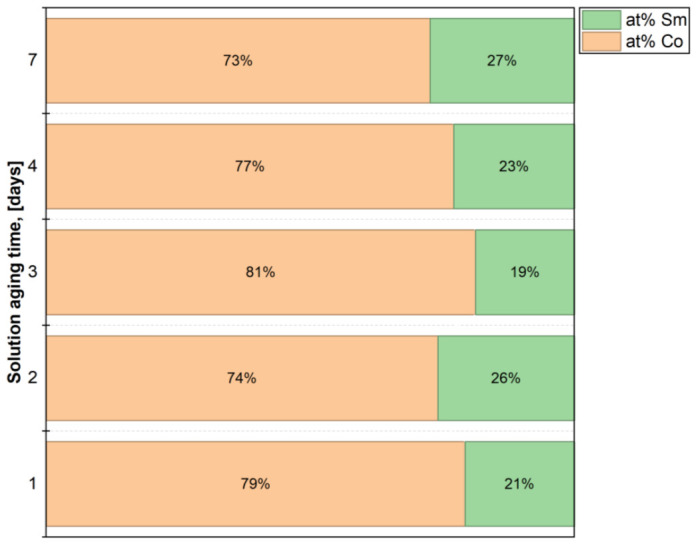
Composition of the alloy deposited from the solution aged with glycine as a complexing agent [at.%].

The deposit was analyzed for chemical composition. The results of cobalt and samarium content in the deposits obtained from solutions aged for 1, 2, 3, 4 and 7 days are shown in Figure 24:

On the first day after preparing the solution, the samarium content in the precipitate is approximately 21 at.%. On the second day of aging, the samarium content increases to 26 at.%. On the third day, it decreases to 19 at.%. In the subsequent stages (days 4 and 7), the samarium content increases again, reaching a maximum of 27 at.% at the longest aging time. The samarium content ranges from 19 at.% to 27 at.%. The chemical composition of the precipitate in the solution aged with glycine is more stable than in the solution with L-arginine. In this atomic system, the difference between the lowest and highest values is 9 at.%, while in the previous case of L-arginine, the variation was much greater, reaching 20 at.%. This confirms that the glycine-based electrolyte maintains greater stability over time.

**Figure 25:**
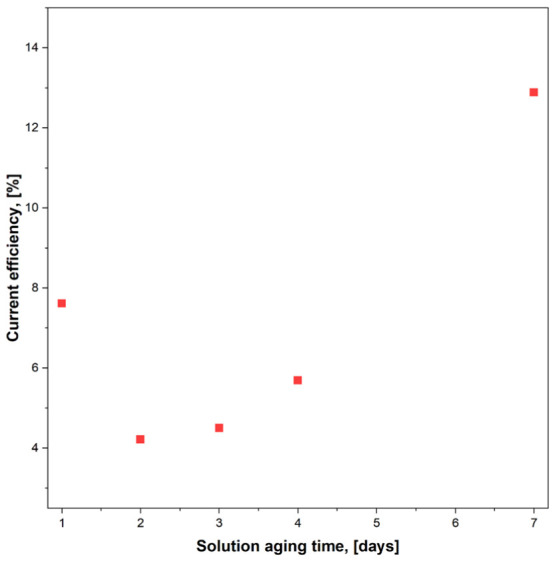
Change in current efficiency depending on the solution aging time for deposition from a solution containing glycine. The red squares represent experimental data points.

The stability of the tested electrochemical system was assessed by determining the effect of the solution aging time on its ability to effectively transfer charge, which was shown in the graph of the change in current efficiency as a function of aging time (Figure 25).

On the first day after solution preparation, the system’s current efficiency is approximately 8%. On the second day of aging, there is a significant drop to approximately 4%, where the efficiency reaches its minimum. Unlike the previous study, subsequent days of aging (days 3 and 4) gradually increase the process efficiency to 4.5% and 6%, respectively. This process accelerates in the final phase—after seven days of electrolyte aging, the current efficiency reaches its maximum at approximately 13%.

**Figure 26:**
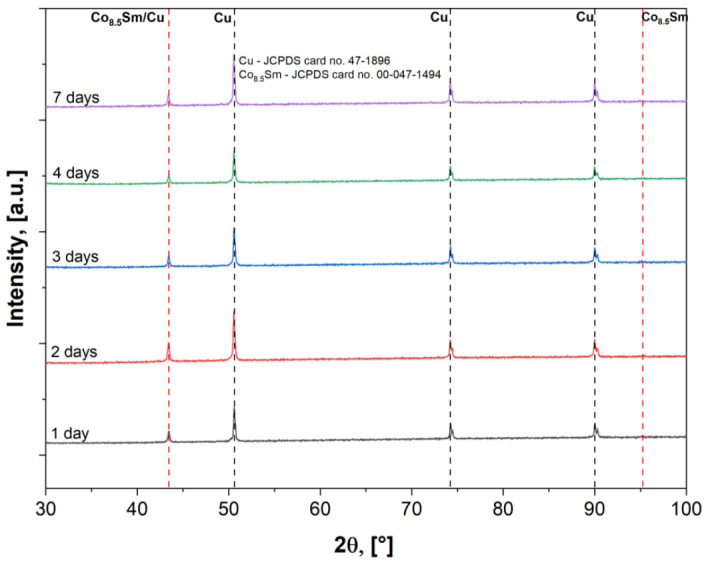
XRD analysis of the electrodeposited layer from the aged solution with glycine as a complexing agent.

The diffraction analysis for a series of samples obtained from the aged glycine solution is presented below (Figure 26):

The obtained XRD profiles remain consistent with results for both freshly prepared and L-arginine-aged solutions. Regardless of the electrolyte aging time, all peaks appear at the same diffraction angles of 44°, 50°, 74°, 90°, and 95°. The signals from the copper substrate (JCPDS card No. 47-1896) continue to dominate at 50°, 74°, and 90°. The Co_8.5_Sm intermetallic phase (JCPDS card No. 00-047-1494) is identified by reflections at 44° and 95°.

## 4. Discussion

Analysis of the obtained electrodeposition results for Sm-Co coatings confirms that the process follows a co-deposition mechanism, in which the reduction of samarium ions occurs with the simultaneous precipitation of a metal from the iron group, in this case, cobalt. Based on studies on the electrochemical preparation of samarium–cobalt alloy coatings using the chronoamperometric method, it was demonstrated that a key aspect enabling the synthesis of these systems from aqueous solutions is the use of appropriate complexing agents, such as L-arginine or glycine, which prevent premature precipitation of samarium oxide Sm_2_O_3_. A higher mass increase was noted in the system with glycine, i.e., 0.0225 g, and for L-arginine at 0.01 g at −1.4 V. The effectiveness of L-arginine results from its specific molecular structure and stronger affinity to form stable complexes with lanthanide ions, which inhibits the process of hydrolysis and precipitation of samarium oxide Sm_2_O_3_ [64]. This phenomenon is important for the process’s repeatability, as confirmed by electrolyte aging studies—the lack of changes in the structure of the resulting precipitate over time indicates the high durability of the developed chemical system, a desirable parameter in potential industrial applications. Interpreting the chemical composition results in correlation with the applied potential allows for the precise determination of the process’s technological window. A potential threshold of −1.4 V was identified, below which a significant increase in the samarium content in the alloy occurs, reaching a maximum of 29 at.% for L-arginine and 35 at.% for glycine at −1.8 V. EDS analysis showed the carbon content in the deposited layers, especially in the L-arginine system (up to 41 at.%). This observation shows that the amino acids used in the electrolyte serve not only as complexing agents but also as a carbon source. Although amino acids act as carbon precursors, the exceptionally high carbon content (up to 41 at.%) observed in some samples may be due to carbon in the electrolyte resulting from insufficient sample rinsing or contamination from sample transportation. Carbon incorporation may be beneficial, as literature suggests it can improve the corrosion and chemical stability properties of coatings. The incorporation of carbon into the Sm-Co structure is critical for enhancing its mechanical and protective performance. Carbon enhances the corrosion resistance and chemical stability of the coatings, which is particularly beneficial in mitigating the degradation caused by hydrogen evolution [65]. Obtaining coatings with a very high samarium content compared to the literature data is a success from a chemical perspective; however, SEM morphological analysis reveals a compromise between the composition and quality of the obtained layers [17,66,67,68]. At the same time, SEM morphological analysis indicated significant process limitations related to the intense co-evolution of hydrogen at low potentials. The accompanying co-evolution of hydrogen leads to a local increase in pH at the electrode surface and the generation of internal stresses. Continuous or less fragmented structures are observed at more positive potentials. Heavily cracked morphologies can be observed at more negative potentials, i.e., from −1.4 V to −1.8 V. Hydrogen embrittlement is a phenomenon limiting the adhesion of the deposited alloy. Furthermore, the scientific implications of the metallic substrate itself must be considered; the significant lattice mismatch between the face-centered cubic Cu substrate and the synthesized intermetallic phase inevitably generates interfacial stresses. These substrate-induced interfacial stresses are distinct from, and additive to, the aforementioned hydrogen-induced internal stresses, acting as another major contributing factor to the prominent crack formation observed under SEM. A significant result of the study is the confirmation of the presence of the Co_8.5_Sm intermetallic phase using XRD. The lack of reflections from pure metals suggests that the process occurs in a controlled manner, leading directly to the formation of an alloy phase. This phase is formed directly during the electrodeposition process without the need for high-temperature heat treatment, indicating the high surface activity of the copper substrate and appropriately selected process parameters. The discrepancies between XPS, XRD, and EDS results are physically consistent and are due to the different probing depths of these techniques. XPS probes only the outermost 5 nm. The increase in samarium on the surface is attributed to its high oxophilicity and preferential surface segregation upon exposure to air, leading to a layer dominated by samarium and cobalt oxides and hydroxides. XPS analysis revealed a surface Co:Sm atomic ratio of approximately 2.3:1 for the L-arginine-stabilized sample, indicating a significant quantitative discrepancy compared to the bulk Co_8.5_Sm stoichiometry (~8.5:1) suggested by the XRD data. This discrepancy is essentially due to the different characteristics and sampling depth of the two characterization techniques. While XRD probes the crystal structure in the bulk deposited layer (several micrometers deep), XPS is a purely surface-sensitive method, recording data only from the top 5–10 nm layer. The pronounced surface enrichment of samarium is attributed to its exceptionally high oxophilicity and preferential surface segregation upon exposure to ambient air [69,70,71]. Rare-earth elements are known to migrate towards the outermost interface, forming thermodynamically stable passivating layers of oxides and hydroxides upon exposure to atmospheric oxygen, leaving the subsurface region relatively depleted in rare-earth elements and enriched in transition metal [72,73,74].

Another significant discrepancy is observed between compositional results obtained by XRF and EDS analysis; for example, at a voltage of −1.8 V in the L-arginine system, XRF records 29 at.% Sm, while EDS indicates approximately 10 at.% Sm. This marked difference is a consequence of the different sampling depths and mechanisms of the two analytical methods. XRF uses high-energy X-ray beams capable of deeply penetrating the entire thickness of the electrodeposited layer and reaching the underlying copper substrate, thus providing an integrated, volumetric composition of the coating. In contrast, the electron beam used in EDS characterization is characterized by a much shallower interaction volume and penetration depth (typically limited to 1–2 µm within these matrices). Because the outermost surface is subject to intense oxidation-induced samarium migration (as confirmed by XPS), the immediate subsurface area examined by EDS appears lower in samarium and higher in cobalt compared to the actual average total volume captured by XRF.

In contrast, XRD integrates the signal from the entire coating thickness, thus reflecting the intermetallic stoichiometry, while EDS provides data from an intermediate depth ratios. Too small a thickness of the deposited layers is insufficient to obtain sharp, clearly visible peaks, which results in a weak signal for the Co_8.5_Sm phase. The results indicate that although it is possible to obtain coatings with very high samarium content, from the point of view of the durability of the obtained coating and mechanical properties, it is crucial to control the potential to minimize the parasitic hydrogen evolution reaction, which is the main barrier to obtaining homogeneous Sm-Co layers. To overcome these limitations, future research will focus on well-defined electrochemical and metallurgical strategies to optimize both the deposition process and the coatings’ functional properties. Pulsed or reverse pulsed electrodeposition techniques will be systematically investigated as the primary means of limiting intense hydrogen co-evolution and minimizing internal stresses in the layers. Post-deposition thermal annealing in an inert atmosphere will be performed to enhance phase homogeneity and potentially improve magnetic properties. Quantitative stress measurements—such as X-ray diffraction peak shift analysis or substrate curvature methods—will be introduced to precisely assess the mechanical state and structural integrity of the deposits. Alternative substrate preparation methods, such as mechanical polishing or electropolishing, will be investigated to minimize interface-induced stresses and mitigate crack initiation. Comprehensive magnetic characterization will be performed as an essential next step to validate the relevance and functionality of the synthesized intermetallic phase.

## 5. Conclusions

Based on the measurements and analysis of the results, it was determined that the use of glycine in the electrodeposition process allows for higher efficiency and a greater mass gain, reaching a maximum of 0.0225 g at a potential of −1.3 V, compared to the system with L-arginine, where the highest recorded value was 0.01 g at −1.4 V. A potential threshold was observed at −1.4 V vs. Ag/AgCl, below which a clear increase in the samarium content in the deposited coating occurs. The highest share of this element was obtained at the lowest investigated potential, −1.8 V, with the addition of glycine favoring a higher concentration of Sm 35 at.% than in the case of L-arginine 29 at.%. It should be emphasized, however, that increasing the samarium content in the alloy is associated with a significant decrease in current efficiency, which is a direct result of the intense, parallel process of hydrogen evolution. XRD structural studies confirmed the successful synthesis of the Co_8.5_Sm intermetallic phase, while demonstrating that the type of amino acid used determines the layer morphology: L-arginine promotes the formation of continuous coatings, whereas glycine induces spherical and agglomerated growth. Surface composition analysis via the XPS method revealed a dominant presence of cobalt and samarium oxides and hydroxides, with an absence of the metallic form in the surface layer. In the system with arginine, the content of cobalt and samarium ions was 13.7 at.% and 6 at.%, respectively, while for the system with glycine, these values were at the level of 8.6 at.% Co and 7.8 at.% Sm, which clearly confirms the surface oxidation process of the obtained alloys.

## Figures and Tables

**Table 1:** Electrolytes and parameters of the electrodeposition process of Sm-Co alloys.

Electrolyte	Chemical Composition of the Electrolyte	pH	Potential vs. Ag/AgCl [V]
Electrolyte I	0.05 M Sm_2_O_3_ 0.1 M CoCl_2_·6H_2_O 0.2 M L-arginine	3.2	from −1.1 to −1.8
Electrolyte II	0.05 M Sm_2_O_3_ 0.1 M CoCl_2_·6H_2_O 0.2 M glycine	3.2	from −1.1 to −1.8

**Table 2:** Comparison of reference XRD data from JCPDS card No. 00-047-1494 and experimental for sample electrodeposited with potential −1.5 V.

2θ from JCPDS Card 00-047-1494 [°]	d [Å]	Measured Sample [°]	d [Å]
43.41	2.62	43.40	2.08
95.41	1.31	95.22	1.04

**Table 3:** EDS analysis results for layers obtained from L-arginine solution [at.%].

Potential vs. Ag/AgCl [V]	Chemical Composition [at.%]			
	Sm	Co	O	C
−1.1	-	14	46	41
−1.2	-	-	-	-
−1.3	-	-	-	-
−1.4	10	16	60	14
−1.5	8	13	56	23
−1.6	12	13	61	14
−1.7	12	9	65	14
−1.8	10	13	64	13

**Table 4:** XPS analysis results for layers obtained from L-arginine solution [at.%].

	O			Cl	Co	Sm
**Binding energy [eV]**	529.4	531.3	533.0	198.1	780.8	1083.5
**Compound/Chemical state**	O-Me	O-Me O=C -OH	O-C -OH	Cl^−^	Co^2+^	Sm^3+^
**Content [at.%]**	2.4	56.6	18.2	3.1	13.7	6.0

**Table 5:** EDS analysis results for layers obtained from glycine solution [at.%].

Potential vs. Ag/AgCl [V]	Chemical Composition [at.%]			
	Sm	Co	O	C
−1.1	5	51	33	11
−1.2	3	77	15	5
−1.3	3	74	17	6
−1.4	4	48	34	14
−1.5	11	11	66	12
−1.6	11	11	64	14
−1.7	10	16	60	14
−1.8	10	10	67	13

**Table 6:** XPS analysis results for layers obtained from glycine solution [at.%].

	O			Cl	Co	Sm
**Binding energy [eV]**	529.4	531.3	533.0	198.1	780.8	1083.5
**Compound/Chemical state**	O-Me	O-Me O=C -OH	O-C -OH	Cl^−^	Co^2+^	Sm^3+^
**Content [at.%]**	5.1	62.3	14.1	2.0	8.6	7.8

## Data Availability

The original contributions presented in this study are included in the article. Further inquiries can be directed to the corresponding author.
